# Fat Infiltration in Eyelids: An Uncommon Clinical Case with Long-Term Monitoring

**DOI:** 10.22336/rjo.2026.20

**Published:** 2026

**Authors:** Maria Amparo Mora Villate, Jhon Alexander Cortes Tribaldos

**Affiliations:** 1 National University of Colombia, Faculty of Medicine, Bogotá, Colombia

**Keywords:** eyelid swelling, fatty infiltration, enophthalmos, blepharoplasty, orbital imaging, MRI = Magnetic Resonance Imaging, HIPAA = Health Insurance Portability and Accountability Act, OD = Oculus Dexter (right eye), HIV = Human Immunodeficiency Virus

## Abstract

**Objective:**

To describe a rare case of fatty tissue infiltration in the eyelids, leading to enophthalmos, and its successful surgical management with long-term follow-up.

**Methods:**

We present the case of a 32-year-old woman with a three-year history of progressive, painless swelling of the right upper and lower eyelids. Clinical and radiologic evaluation included physical examination and magnetic resonance imaging (MRI). Histopathological analysis confirmed the diagnosis. Surgical resection was performed, and the patient was followed for over 24 months.

**Results:**

Examination revealed increased soft tissue volume in the right eyelids, narrowing of the palpebral fissure, and subtle hyperpigmentation. MRI showed fatty infiltration of the eyelids and reduced intraconal orbital fat, resulting in right-sided enophthalmos. No systemic, autoimmune, or infectious causes were identified. Histopathology confirmed fatty tissue infiltration and excluded neoplastic processes. Surgical excision of the abnormal tissue via upper and lower eyelid blepharoplasty was performed. At 14-month follow-up, the patient showed no signs of recurrence and was satisfied with the functional and cosmetic outcome, although enophthalmos persisted.

**Discussion:**

No instances of fatty tissue infiltration in the eyelid were discovered despite a thorough search for comparable cases in the literature. A few examples of lipomas affecting just one eyelid have been documented. But unlike a lipoma, this fat infiltration lacks fibrous septa and is poorly confined.

**Conclusions:**

Fatty infiltration of the eyelids is an exceptionally rare presentation, especially when accompanied by intraconal fat loss and enophthalmos. This case highlights the importance of thorough imaging and histological analysis in atypical eyelid swelling. Surgical resection may offer a definitive and lasting solution. To our knowledge, this is the first reported case of its kind.

## Introduction

Eyelids can be affected by various local and systemic conditions, such as autoimmune diseases, neoplasms, heart failure, and nephropathies, which can present with multiple manifestations in the orbital region. Progressive and painless eyelid swelling may be caused by an edematous syndrome or a mass [1]. Among the most common neoplastic pathologies is lymphoma, which generally presents as firm masses and is associated with exophthalmos [2,3].

On the other hand, lipomas are benign tumors composed of adipose tissue. They are rare and must be differentiated from herniated orbital fat, cystic lesions, and the lacrimal gland. Lipomas are most commonly found subcutaneously on the torso, neck, and extremities. However, all areas can be affected by this type of lesion, which is histologically characterized by fatty lobules separated by fibrous septa. If there is any doubt, magnetic resonance imaging should be performed to evaluate possible orbital involvement and plan the appropriate surgical procedure [4,5].

This case report describes a rare case of fatty tissue infiltration of the eyelids in a 32-year-old female patient. The report includes details of the clinical presentation, diagnostic approach, and surgical management. The methods adhered to ethical guidelines outlined in the Declaration of Helsinki and complied with HIPAA regulations.

## Case presentation

**Fig. 1:**
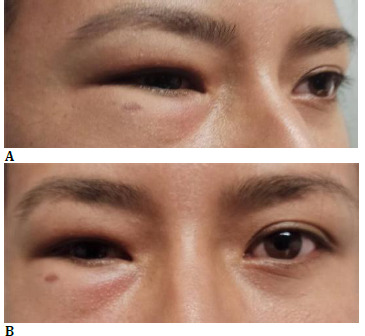
**A, B** Initial appearance with eyelid asymmetry and swelling

**Fig. 2:**
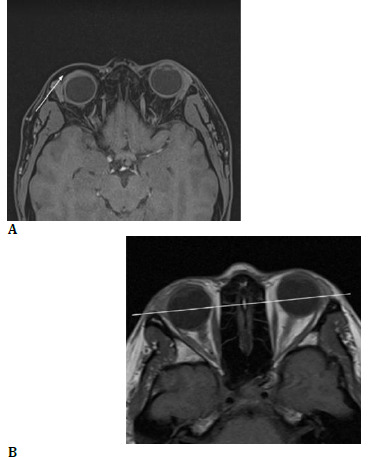
**A, B** Orbital MRI showing eyelid fat infiltration, intraconal fat loss, and enophthalmos

A 32-year-old female patient with no significant medical history was referred to oculoplastics in 2021 for a clinical case with a 3-year history of progressive swelling of the upper and lower eyelids of the right eye, reducing the size of the palpebral fissure. The ophthalmological exam showed increased soft tissue volume in the upper and lower eyelids, no palpable masses or lacrimal gland, a reduced palpebral fissure, and slight darkening of the right eyelid skin. Eye movements were normal, pupils were reactive, and corrected visual acuity was 20/20 (**Fig. 1A, B**). The anterior segment and intraocular pressure were normal. An orbital MRI revealed fatty tissue infiltration in the upper and lower eyelids, reduced fat in the intraconal space, and right eye enophthalmos (**Fig. 2A, B**). Internal medicine assessments excluded storage diseases, autoimmune disorders, infections (human immunodeficiency virus [HIV] and tuberculosis), and thyroid disease. Allergy evaluations ruled out allergic conditions.

In the presence of fat-like tissue and the need for histological confirmation, a biopsy of the tissue infiltrating the eyelids was performed, with immunohistochemical markers requested. Pathology confirmed the presence of fatty tissue and ruled out lymphoma or other neoplastic pathology.

**Fig. 3:**
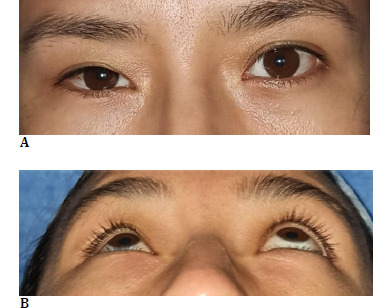
**A, B** Appearance following upper and lower eyelid surgery

**Fig. 4:**
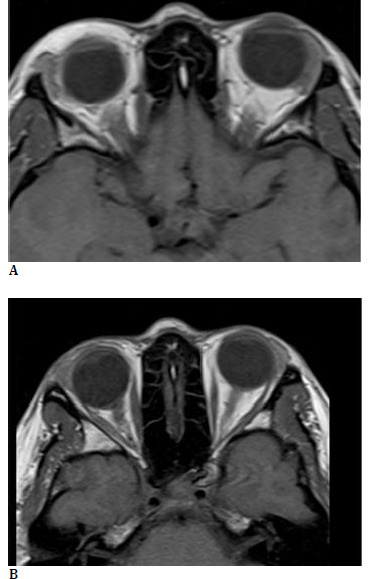
**A, B** Long-term follow-up with no recurrence

Surgical management was decided upon, involving resection of fatty tissue from the upper and lower eyelids, with an approach similar to that used in upper and lower blepharoplasty. The resected tissue was sent for biopsy, which reported adipose tissue. The patient was followed for 24 months post-surgery without any clinical or imaging evidence of recurrence of fatty tissue infiltration. Enophthalmos of the right eye persisted, but the patient reported no discomfort (**Figs. 3A, B; 4A, B**).

## Discussion

Despite an exhaustive search for similar cases in the scientific literature, no cases of fatty tissue infiltration in the eyelid were found. There are a few reported cases of lipomas affecting a single eyelid. However, the characteristics of this fat infiltration differ from those of a lipoma: it is not well circumscribed and lacks fibrous septa.

Magnetic resonance imaging (MRI) was crucial for identifying fat infiltration and reduced intraconal fat, resulting in significant enophthalmos in the right eye (OD). This finding, along with the absence of systemic disease, lymphoma-type neoplasm, allergic, or infectious conditions, guided the decision towards surgical management. The underlying pathophysiological mechanism responsible for the observed reduction in intraconal fat, despite palpebral fatty infiltration, remains unclear. Further investigation is needed to elucidate the relationship between these phenomena.

The surgical approach, similar to a blepharoplasty, allowed resection of the abnormal fatty tissue and provided the patient with aesthetic and functional relief. Histopathological confirmation of fat infiltration validated the surgical decision.

As this case represents an unreported condition with an unclear pathophysiological basis, long-term follow-up is essential to monitor its behavior and progression. Further observation will be necessary to understand its implications and potential changes over time.

This case highlights the need to consider unusual diagnoses in patients with atypical clinical presentations. It demonstrates that surgical resection can be an effective and durable treatment for eyelid fat infiltration.

## Conclusion

This case represents an unusual presentation of palpebral fat infiltration associated with intraconal fat reduction and enophthalmos, a combination not previously described in the literature. Comprehensive clinical evaluation and orbital imaging were essential for diagnosis. Surgical resection via a blepharoplasty-like approach resulted in good cosmetic and functional outcomes, with no recurrence at 14-month follow-up. This report underscores the importance of considering rare etiologies in persistent eyelid swelling and the value of individualized surgical management.
